# An Open Dataset for Wearable SSVEP-Based Brain-Computer Interfaces

**DOI:** 10.3390/s21041256

**Published:** 2021-02-10

**Authors:** Fangkun Zhu, Lu Jiang, Guoya Dong, Xiaorong Gao, Yijun Wang

**Affiliations:** 1 State Key Laboratory of Reliability and Intelligence of Electrical Equipment, Tianjin Key Laboratory of Bioelectromagnetic Technology and Intelligent Health, Hebei University of Technology, Tianjin 300132, China; 201831404017@stu.hebut.edu.cn; 2 State Key Laboratory on Integrated Optoelectronics, Institute of Semiconductors, Chinese Academy of Sciences, Beijing 100083, China; jianglu@semi.ac.cn; 3 University of Chinese Academy of Sciences, Beijing 100049, China; 4 School of Medicine, Tsinghua University, Beijing 100084, China; gxr-dea@tsinghua.edu.cn

**Keywords:** brain-computer interface (BCI), steady-state visual evoked potential (SSVEP), electroencephalogram (EEG), dry electrode, open dataset, wearable BCI

## Abstract

Brain-computer interfaces (BCIs) provide humans a new communication channel by encoding and decoding brain activities. Steady-state visual evoked potential (SSVEP)-based BCI stands out among many BCI paradigms because of its non-invasiveness, little user training, and high information transfer rate (ITR). However, the use of conductive gel and bulky hardware in the traditional Electroencephalogram (EEG) method hinder the application of SSVEP-based BCIs. Besides, continuous visual stimulation in long time use will lead to visual fatigue and pose a new challenge to the practical application. This study provides an open dataset, which is collected based on a wearable SSVEP-based BCI system, and comprehensively compares the SSVEP data obtained by wet and dry electrodes. The dataset consists of 8-channel EEG data from 102 healthy subjects performing a 12-target SSVEP-based BCI task. For each subject, 10 consecutive blocks were recorded using wet and dry electrodes, respectively. The dataset can be used to investigate the performance of wet and dry electrodes in SSVEP-based BCIs. Besides, the dataset provides sufficient data for developing new target identification algorithms to improve the performance of wearable SSVEP-based BCIs.

## 1. Introduction

Brain computer interface (BCI) constructs a direct communication channel between the brain and external devices by coding and decoding brain activities (mainly physiological electrical signals) [1,2]. Recently, electroencephalogram (EEG)-based BCIs have gradually moved from the laboratory to the public’s vision, and there are more and more application scenarios such as diagnosis, rehabilitation, disability assistance and fatigue monitoring [3,4,5,6,7,8]. Among various applications, steady-state visual evoked potential (SSVEP)-based BCI has attracted more and more attention on account of its high information transfer rate (ITR), less user training, and easy operation [5,9,10,11]. Researchers have developed many encoding and decoding algorithms to improve system performance [12,13,14,15,16,17,18,19,20,21,22,23]. To facilitate the evaluation of the performance of algorithms, open datasets for SSVEP-based BCIs have emerged in recent years [24,25,26]. High efficiency of these open datasets has benefited the studies in high-speed BCI spellers for researchers. However, to improve the practicality of SSVEP-based BCIs, a wearable BCI system is in great demand. Compared with the BCI system, which uses a traditional bulky, expensive, and wired EEG system, a wearable BCI system is more preferable in practice due to its advantages such as portability, mobility, easy preparation, and low cost. However, in more complex environments, practical applications of wearable BCI systems face more challenges in data acquisition, data analysis, and user experience. As far as we know, a public dataset with a large number of subjects for a wearable SSVEP-based BCI is still missing.

An EEG electrode is an important factor in the application of a wearable BCI system. Wet electrodes and dry electrodes are two types of scalp electrodes that are frequently used to obtain EEG signals. Wet electrodes have better signal quality and are more comfortable to wear [27]. However, the preparation of the wet electrodes before the experiment requires professional technical support and cleaning the conductive paste after use is also time-consuming. Besides, wet electrodes cannot be used for a long duration recording as the gel will dry out over a period of time. The dry electrode does not need conductive paste and provides a convenient and durable way for EEG acquisition [28]. Besides, the dry electrode is suitable for making high-density electrode arrays that collect EEG signals with high spatial resolution [29]. However, the signal quality and user experience of dry electrodes are generally worse due to the tight press of electrodes onto the scalp. Although many studies have compared the difference between wet and dry electrodes in EEG recording and BCI applications [30,31,32,33]. A comprehensive comparison of BCI performance and user experience between the two types of electrodes is still missing for a wearable SSVEP-based BCI.

Time-variant effects of user experience and system performance during long-duration system use requires careful investigation when moving the SSVEP-based BCI from the laboratory experiments to real-life scenes. For example, fatigue caused by continuous visual stimulation can lead to user’s discomfort and degrade the system performance [8]. For a longer period of system operation, most users will feel fatigue, drowsiness, difficulty in concentration and other dissatisfaction or even uncomfortable feelings to flickering stimulus [7]. Self-reported questionnaires and EEG indices are commonly used to explore the effect of fatigue on EEG and BCI performance [34,35]. In addition to fatigue, user experience regarding the comfort level of the electrode is also crucial for long time system use. A tradeoff between signal quality, convenience, and user comfort has to be made in real practice. Currently, in long-time system use, time-variant effects of user experience and system performance for a wearable SSVEP-based BCI still remains unknown.

By considering the major factors described above, this study constructs an open dataset with a large number of subjects for a wearable SSVEP-based BCI towards practical applications. Compared with the existing datasets, the present dataset has the following characteristics: (1) An 8-channel wireless EEG headband was used to record SSVEPs from the occipital region; (2) a total of 102 subjects were recorded using the same 12-target SSVEP-based BCI paradigm; (3) dry and wet electrodes were used separately for each subject, and the electrode impedance was measured; (4) a total of 20 blocks of data were recorded across a long operation time of around 2 h; (5) user experience questionnaires on wet and dry electrodes were collected. Due to these abovementioned features, the present dataset is especially useful for developing and evaluating SSVEP detection algorithms for a wearable BCI.

The rest of this paper is structured as follows. Section 2 expounds the materials and methods including the information of experimental support, participants, stimulus presentation, EEG device and electrodes, experiment protocol, data acquisition, data preprocessing, performance evaluation, and data records. Section 3 illustrates the results of EEG characteristics, BCI performance, user experience questionnaire, and the effects of electrodes, subjects, and operation time. Section 4 concludes and discusses the future work needed for improving the dataset.

## 2. Materials and Methods

### 2.1. Experimental Support

This study relied on the BCI Brain-Controlled Robot Contest at the 2020 World Robot Contest to recruit participants. All participants were informed before the experiment that the data would be used for non-commercial scientific research. All subjects met the following criteria: (1) No history of seizures or other mental illnesses; (2) no attention deficit or hyperactivity; (3) no history of brain injury or intracranial implantation.

### 2.2. Participants

One hundred and two healthy subjects (64 males and 38 females, with an average age of 30.03 ± 0.79 years ranging from 8 to 52 years) with normal or corrected-to-normal eyesight participated in the experiment. All subjects used SSVEP-based BCI for the first time and were required to read and sign an informed consent form at the beginning of the experiment. Each participant was required to fill out a questionnaire about the comfort of each electrode after the experiment. The study was conducted in accordance with the Declaration of Helsinki, and the protocol was approved by the Research Ethics Committee of Tsinghua University (N0.20200020).

### 2.3. Stimulus Presentation

**Figure 1:**
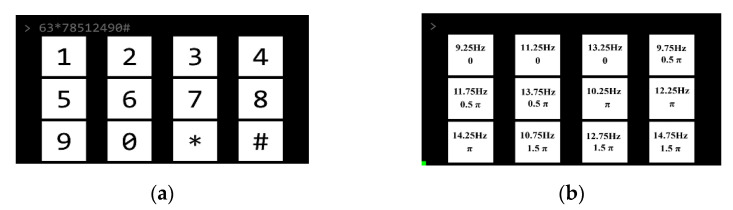
(**a**) Stimulation interface of the 12-target brain-computer interface (BCI) speller. (**b**) Frequency and phase values for all targets.

This research designed an online BCI system with a 12-target speller as a virtual keypad of a phone (see Figure 1). As shown in Figure 1a, a 3×4 stimulus matrix was displayed on a 27-inch LCD display (DELL S2719H; resolution, 1920×1080 pixels; refresh rate, 60 Hz), being viewed from a distance of 60 cm. The size of each stimulation target was 288×288 pixels ($8.5^{{}^{\circ}}\times 8.5^{{}^{\circ}}$), the size of the entire stimulation matrix area was 1860×945 pixels ($51.5^{{}^{\circ}}\times 27.5{}^{\circ}$), and the vertical and horizontal distances between two adjacent stimuli were 20 and 141 pixels, respectively. The stimulation was completed by Psychophysics Toolbox Ver.3 (PTB-3) in MATLAB (Mathworks, Inc. Natick, Massachusetts, United States) [36].

The visual flickers of the speller were coded using the joint frequency and phase modulation (JFPM) method [5]. Twelve frequencies (9.25–14.75 Hz with a 0.5 Hz interval) were used and the phase difference between two adjacent frequencies was 0.5π. As shown in Figure 1b, the locations of 12 stimulations were selected to avoid neighboring frequencies at adjacent stimulus locations. Visual flickers were presented on the screen in the light of the sampling sinusoidal stimulation method [11]. Theoretically, the stimulus sequence s(f,ϕ,i) at frequency f and phase ϕ is generated by:

(1) $$s(f,\phi ,i)=\frac{1}{2}\{1+sin[2\pi f(i/\mathrm{RefreshRate})+\phi ]\},$$

where sin() generates a sequence of sine waves, i is the frame index, f and ϕ are the frequency and phase for the target, respectively, and RefreshRate is set to be 60, where 0 and 1 represent the lowest and highest luminance in the stimulation sequence, respectively.

### 2.4. EEG Device and Electrodes

**Figure 2:**
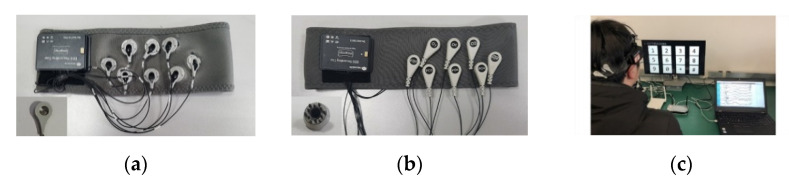
The 8-channel wireless EEG headband with (**a**) wet and (**b**) dry electrodes. (**c**) A subject wearing the headband in the 12-target BCI experiment.

An 8-channel NeuSenW (Neuracle, Ltd. Changzhou, China) wireless EEG acquisition system was used to record the SSVEPs in this study. The dry and wet electrodes were used to make wearable EEG headbands. Figure 2a,b shows the wet and dry electrode headbands. For the dry-electrode headband, dry multi-pin electrodes (Florida Research Instruments, Inc. Florida, United States) were used for recording SSVEPs and two conductive fabric electrodes were used as reference and ground electrodes on the forehead. Both headbands were elastic and come in three sizes: large, medium, and small, which can perfectly fit various head shapes, ensuring the quality of the EEG signals as well as the comfort of users.

Each subject was required to wear the two headbands to record data for comparison. The wearing order of the two headbands were randomized. In total, 53 subjects wore the dry-electrode headband first and 49 subjects wore the wet-electrode headband first. Because the conductive paste was applied to the wet electrodes, participants who wore the wet-electrode headband first washed and dried their hair before wearing the dry-electrode headband.

### 2.5. Experimental Protocol

**Figure 3:**
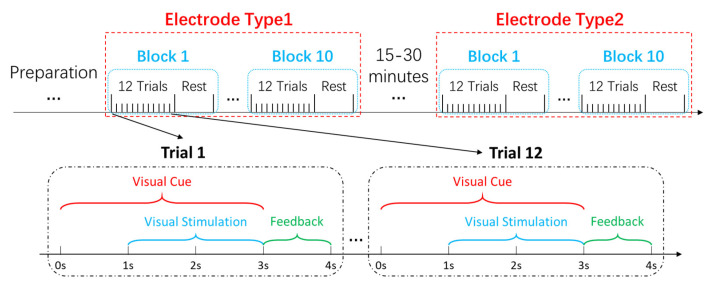
The schematic diagram of the experimental design for each subject.

In this study, an online 12-target BCI experiment was designed using the cue-guided target selecting task. Figure 3 illustrates the experimental procedures for each subject. Ten consecutive blocks of data were recorded for each type of electrodes. Each block included 12 trials, and each trial corresponded to each target. At the beginning of each block, a random sequence of the 12 characters appeared at the top of the screen. Each trial’s stimulation began with a visual cue (a red square that lasted for 1 s). Subjects were instructed to follow the prompts to stare at the corresponding target and avoid blinking their eyes for the next 2 s when all the targets started flashing, while a synchronized trigger signal was recorded. The cue remained in the flickering duration. Each trial ended with a rest time of 1 s, in which the visual feedback (i.e., typed keys) calculated by an online filter bank canonical correlation analysis (FBCCA) method [13], which was presented in the input field. Subjects rested for several minutes after a block to avoid fatigue.

In the experiments, the participants continued to perform a 40-target spelling task for 2 to 6 blocks (data not presented in this study) after the 12-target task. After that, they wore another headband and repeated the same tasks. The interval between the 12-target spelling tasks with the two headbands was 15 to 30 min. In total, the participants continued to use the SSVEP-based BCI in a duration of around 2 h in a laboratory without electromagnetic shielding and sound insulation.

### 2.6. Data Acquisition

EEG data were recorded using Neuracle EEG Recorder NeuSen W (Neuracle, Ltd.), a wireless EEG acquisition system with a sampling rate of 1000 Hz. Eight electrodes (POz, PO3, PO4, PO5, PO6, Oz, O1 and O2, sorted by channel index in the dataset) were placed at the parietal and occipital regions on the basis of the international 10 to 20 system to record SSVEPs and two electrodes were placed at the forehead as the reference and ground, respectively. Stimulus events generated by the stimulus program synchronized to the EEG data were recorded on an event channel. The event channel of EEG data recorded the stimulation events generated by the stimulation program.

### 2.7. Data Preprocessing

In accordance with the stimulus onsets recorded in the event channel of the continuous EEG data, data epochs could be extracted. The length of each data epoch was 2.84 s, including 0.5 s before the stimulus onset, 0.14 s for visual response delay, 2 s for stimulus, and 0.2 s after stimulus. With the purpose of reducing the storage and computation costs, all data were down sampled to 250 Hz [13,14].

### 2.8. Performance Evaluation

The performance of SSVEP-BCI was commonly evaluated by classification accuracy and ITR. ITR (in bits/min) can be calculated as follows:

(2) $$ITR=(log_{2}N+Plog_{2}P+(1-P)log_{2}[\frac{1-P}{N-1}])\times 60/T,$$

where N indicates the number of targets, P indicates the averaged classification accuracy of all targets, and T (seconds/selection) indicates the average time taken to complete a selection.

Signal-to-noise ratio (SNR) was wildly used for evaluating the signal quality of SSVEP. Most studies calculated the narrowband SNR (i.e., the spectral amplitude at the stimulus frequency to the mean value of the five neighboring frequencies) as follows:

(3) $$SNR=20\log\frac{y(f)}{\sum_{k=1}^{5}\left[y(f-\Delta f\cdot k)+y(f+\Delta f\cdot k)\right]},$$

where y(f) denotes the amplitude spectrum at frequency f calculated by the Fast Fourier Transform (FFT), and Δf denotes the frequency resolution.

### 2.9. Data Records

#### 2.9.1. EEG Data

The dataset includes 102 MATLAB MAT files corresponding to 102 subjects’ EEG data. All files were named according to the participants’ index (i.e., S001.mat, S102.mat). Each file contained a 5-D matrix named “data” with dimensions of [8, 710, 2, 10, 12] and was stored as double-precision floating-point values. The five dimensions respectively indicated “Channel index”, “Time points”, “Electrode index”, “Block index”, and “Target index”. Each matrix corresponded to 240 epochs (12 targets × 10 blocks × 2 electrodes) and each epoch was composed of raw EEG data without any processing from eight channels with the length of 2.84 s (2.84 s × 250 = 710 time points). A “stimulation_information.pdf” file listed the target index, labels, and the corresponding stimulation parameters (see Figure 1 for frequency and phase).

#### 2.9.2. Subject Information and Questionnaires

The “subjects_information.mat” file listed the information of all 102 subjects together with a questionnaire on the comfort level and preference of the two headbands after the experiment. For each participant, there were 10 columns of parameters (factors). The first 4 columns were the subjects’ personal information including “subject index”, “gender”, “age”, and “dominant hand”. The 6 columns (5th to 10th) were listed as results in questionnaires, which were “Comfort of dry electrode headband”, “Wearing time of dry electrode when pain occurs”, “Comfort of wet electrode headband”, “Wearing time of wet electrode when pain occurs”, “Only consider comfort, headband preference”, and “comprehensively consider comfort and convenience (need assistance from others, conductive paste, shampoo, etc.), headband preference”. The last column showed the order of wearing the two headbands.

#### 2.9.3. Impedance Information

The electrode impedances recorded before each block were provided in the data matrix of “Impedance.mat” with dimensions of [2, 8, 10, 102]. The numbers in the four dimensions represented the number of channels, blocks, headband types (1: wet, 2: dry) and subjects, respectively. The impedance information was useful to find the relationship between impedance and BCI performance.

## 3. Results

### 3.1. EEG Characteristics of SSVEPs

**Figure 4:**
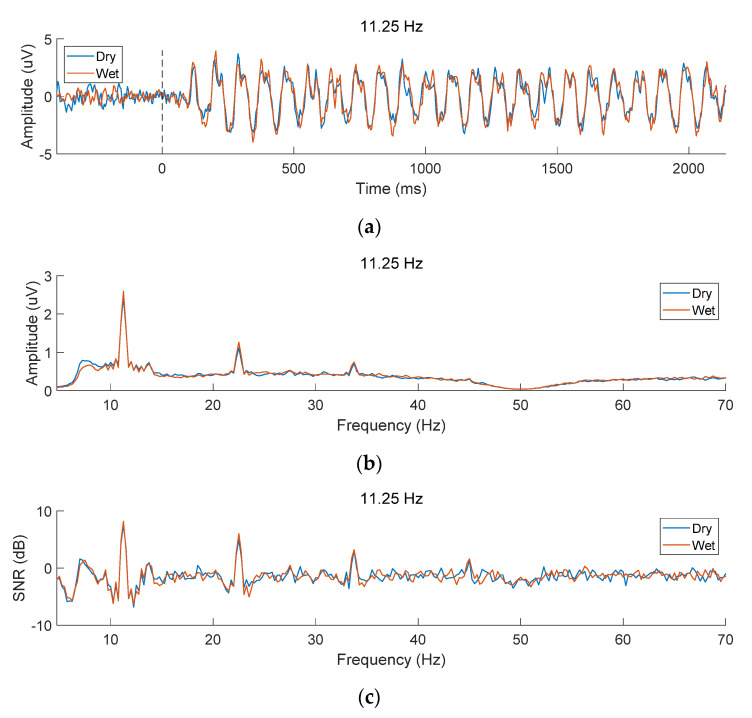
(**a**) Time-domain waveform, (**b**) amplitude spectrum, and (**c**) SNR of EEG signals recorded with dry and wet electrodes. SSVEPs at 11.25 Hz from the Oz channel were averaged across all subjects and all blocks. The black dashed line at horizontal axis 0 in the subfigure (**a**) represents the stimulus onset of the 2 s visual stimulation.

This study used the temporal waveform, amplitude spectrum and *SNR* to compare the data quality of SSVEPs recorded by the two types of electrodes. Figure 4a illustrates the time-domain signal of SSVEP at 11.25 Hz in the Oz channel averaged by all subjects and 10 blocks with dry and wet electrodes, respectively. The data were band-pass filtered at 7.25 to 90 Hz to observe obvious time characteristics of SSVEP [24]. For both types of electrodes, there was a ~140 ms visual latency after the stimulus onset, then a sinusoidal-like SSVEP time-locked to stimulus could be observed [37,38]. The frequency and phase of SSVEP remained stable during the 2 s stimulation time. Figure 4b illustrates the amplitude spectrum with clear peaks at 11.25 Hz and its harmonic frequencies (22.5 Hz, 33.75 Hz, and 45 Hz). As the response frequency increased, the amplitude of the fundamental frequency and harmonic components dropped sharply. The amplitudes of wet and dry electrodes were comparable (wet vs. dry, fundamental: 2.597 μV vs 2.444 μV, second harmonic: 1.262 μV vs 1.119 μV, third harmonic: 0.746 μV vs 0.706 μV, fourth harmonic: 0.316 μV vs 0.305 μV). Paired *t*-tests indicate that the difference is not significant (p>0.05) except for the second harmonic (p<0.05). The SNRs were consistent to the amplitudes.

As shown in Figure 4c, the SNRs gradually changes with increasing frequency, because the background noise in the high frequency band is lower. The SNRs are also comparable between wet and dry electrodes (wet vs. dry, fundamental: 8.17 dB vs. 7.51 dB, second harmonic: 6.01 dB vs. 4.91 dB, third harmonic: 3.19 dB vs. 3.08 dB, fourth harmonic: 1.59 dB vs. 1.35 dB). Paired *t*-tests indicate that the difference is not significant (all p>0.05).

### 3.2. Online BCI Performance

**Figure 5:**
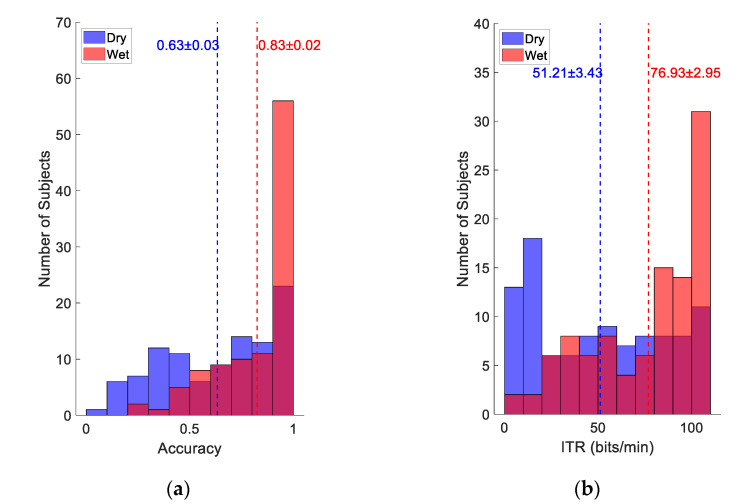
Distribution of number of subjects with regard to (**a**) classification accuracy and (**b**) ITR in the online BCI experiment. The dotted lines represent the average performance of all subjects.

Figure 5 illustrates the distribution of classification accuracy and ITR across subjects in the online BCI experiment. Although SSVEP signals averaged across subjects and trials were comparable in Figure 4, the BCI performance based on single-trial SSVEP classification shows large difference between the wet and dry electrodes. In Figure 5a, 56 subjects achieved classification accuracy above 0.9 with the wet electrodes, whereas only 23 subjects reached 0.9 accuracy with the dry electrodes. On the contrary, the number of subjects with low accuracy below 0.5 for the dry electrodes was much larger than that of the wet electrodes (37 vs. 8). The average classification accuracy of all subjects was 0.83 ± 0.02 for the wet electrode and 0.63 ± 0.03 for the dry electrode. In Figure 5b, ITR had a similar distribution, and the average ITR for wet and dry electrodes was 76.93 ± 2.95 bits/min and 51.21 ± 3.43 bits/min, respectively. Paired *t*-tests indicate that the differences of accuracy and ITR between the dry and wet electrode were both significant (all p<0.001). Note that the stimulation time (2 s) was used as the selection time *T* in the calculation of a theoretical ITR as shown in Equation (2).

### 3.3. Offline Analysis

The FBCCA [13] and the task-related component analysis (TRCA) [14] are two methods that were commonly used to extract SSVEPs in EEG signals. In particular, as a supervised algorithm that requires calibration data, classification accuracy of TRCA was calculated by a leave-one-out cross-validation method. This study adopted FBCCA and filter bank TRCA (FBTRCA) methods in the offline analysis to make better use of the information from fundamental and harmonic frequencies for SSVEP detection. The input signals of the filter bank were composed of the data from all eight electrodes and the data length used for parameter optimization was 1 s after adding a visual delay of 140 ms. Note that, the parameters for the FBCCA method in the online system were adopted from another 12-target SSVEP BCI experiment, which were not optimized with the present paradigm. For the algorithm parameters of online FBCCA, please refer to [13].

#### 3.3.1. FBCCA Method

**Figure 6:**
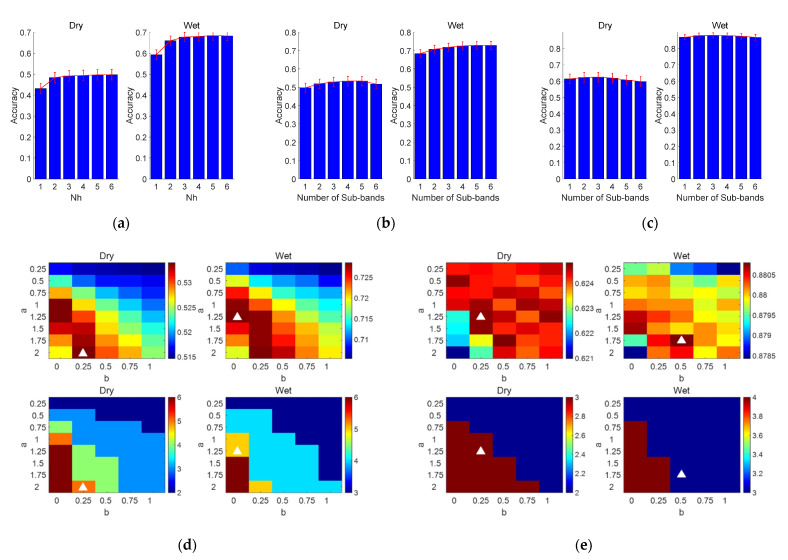
(**a**) In the standard CCA method, the classification accuracy of the different harmonics at 1 s data length. Classification accuracy at different N values (from 1 to 6 with a step of 1): (**b**) FBCCA, (**c**) FBTRCA. The weight vector w (i.e., a and b) and the number of sub-bands N corresponding to the maximum classification accuracy optimized by the grid search method: (**d**) FBCCA, (**e**) FBTRCA. The error bars in (**a**–**c**) represent the standard errors. In (**d**,**e**), the color bar in the top two subgraphs represents the classification accuracy, while the color bar in the bottom two subgraphs corresponds to the number of sub-bands N.

As shown in Figure 6a, the harmonic number Nh of the sinusoidal reference signal in the standard canonical correlation analysis (CCA) process was firstly optimized. The averaged classification accuracy increased with the increase of Nh (dry electrode, Nh=1:0.432, Nh=2:0.483, Nh=3:0.492, Nh=4:0.494, Nh=5:0.497, Nh=6:0.498; wet electrode, Nh=1:0.593, Nh=2:0.660, Nh=3:0.677, Nh=4:0.680, Nh=5:0.684, Nh=6:0.683). These results show that the addition of harmonic components to the input signals are useful to the performance improvement of the standard CCA method. Paired *t*-tests showed that there was a significant difference in classification accuracy between Nh=4 and Nh=5 (dry: p=0.01, wet: p<0.01), but there was no significant difference in classification accuracy between Nh=5 and Nh=6 (dry: p=0.71, wet: p=0.32). Therefore, Nh was set to be 5 for each standard CCA process in the FBCCA method.

For the filter bank method, the number of sub-bands N and the weight vector w of each component were optimized by the grid search method. The weight vector is calculated as follows:

(4) $$w(n)=n^{-a}+b,n\in [1,N],$$

where a, b, and N are limited to [0.25:0.25:2], [0:0.25:1], and [1:1:6], respectively. Figure 6d indicates the maximum classification accuracy and its corresponding parameter values. The optimized parameter values were different for the two types of electrodes (dry: a=2, b=0.25, wet: a=1.25, b=0), and the optimal accuracy was also different (dry: 0.53 ± 0.03, wet = 0.73 ± 0.02). Figure 6b shows the classification accuracy under different N values from 1 to 6. The classification accuracy increased significantly with the increase of the number of sub-bands and reached the peak value at the same N value (N=5).

#### 3.3.2. FBTRCA Method

For FBTRCA, the same grid search method was used to optimize w and N. As shown in Figure 6e,c, parameters and optimal accuracy under the FBTRCA method were also different for the two types of electrodes (dry: a=1.25, b=0.25, N=3, accuracy = 0.62 ± 0.03, wet: a=1.75, b=0.5, N=3, accuracy = 0.88 ± 0.02).

#### 3.3.3. Performance Comparison

**Figure 7:**
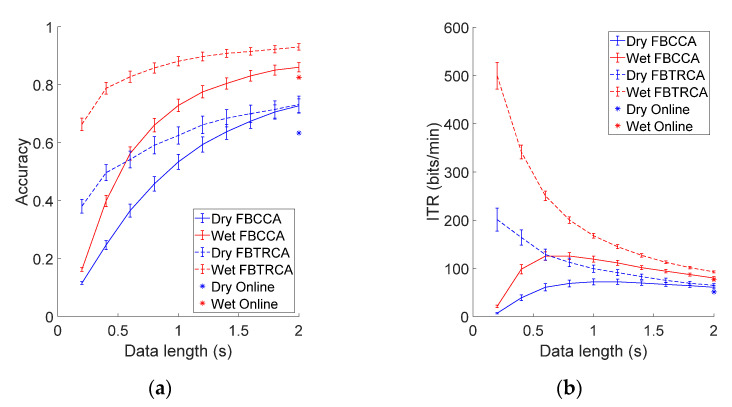
Averaged (**a**) classification accuracy and (**b**) *ITR* across all subjects of the FBCCA method and FBTRCA method with different data length (from 0.2 s to 2 s with a 0.2 s interval) for the dry and wet electrode. The error bars represent standard errors. The asterisk represents the performance of the online system.

This study further compared the classification performance of FBCCA and FBTRCA after offline optimization, and the results of the online system were also included in the comparison (see Figure 7). As shown in Figure 7, the BCI performance of the FBTRCA method outperforms that of the FBCCA method. The employment of individual calibration data in FBTRCA facilitated the detection of SSVEPs. For FBCCA, the classification accuracy was at the lowest at 0.2 s (dry: 0.12 ± 0.01, wet: 0.16 ± 0.01), then increased at longer data length and reached the maximum at 2 s (dry: 0.73 ± 0.02, wet: 0.86 ± 0.02). However, *ITR* first increased and then decreased as the data length increased. The maximum *ITR* of wet and dry electrodes was 125.6 ± 8.88 bits/min (0.8 s) and 72.25 ± 5.48 bits/min (1.2 s) respectively. For FBTRCA, the classification accuracy was the lowest (dry: 0.38 ± 0.02, wet: 0.66 ± 0.02) and the *ITR* was the highest (dry: 201.2 ± 23.87 bits/min, wet: 499.5 ± 27.59 bits/min) with a data length of 0.2 s. When the data length became longer, the accuracy increased and reached the highest at 2 s (dry: 0.73 ± 0.03, wet: 0.93 ± 0.01), but the *ITR* reached the lowest (dry: 64.83 ± 3.74 bits/min, wet: 92.71 ± 2.06 bits/min). In particular, at the data length of 2 s, the performance of both methods was better than the online system (Accuracy, dry: 0.63 ± 0.03, wet: 0.83 ± 0.02; *ITR*, dry: 51.21 ± 3.43 bits/min, wet: 76.93 ± 2.95 bits/min), which indicates that offline optimization can effectively improve classification performance. The comparison also demonstrates the efficacy of using the dataset to evaluate the BCI performance of different SSVEP detection methods.

### 3.4. Individual Difference

**Figure 8:**
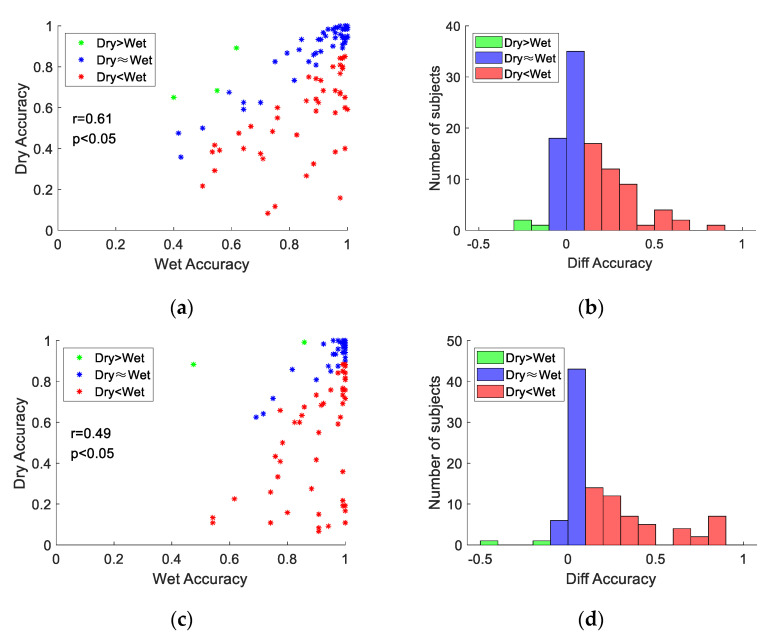
Correlation analysis of the performance between dry and wet electrodes for each subject: (**a**) FBCCA, (**c**) FBTRCA. Distribution of the number of subjects with regard to the accuracy difference between wet and dry electrodes: (**b**) FBCCA, (**d**) FBTRCA.

Figure 8 shows correlation analysis of the performance between dry and wet electrodes. As shown in Figure 8, for both types of electrodes, there was clear individual difference of classification accuracy across subjects. Figure 8a,c illustrates that the overall distribution showed significant positive correlations (FBCCA: r=0.61, p<0.05, FBTRCA: r=0.49, p<0.05) between accuracies for the wet and dry electrodes. These results indicate that the variety of classification accuracy is highly related to the individual difference of SSVEP signals. According to different levels of performance gap between dry and wet electrodes, this study divided all participants into three groups. The first group meant the accuracy of wet electrode was at least 10% higher than that of dry electrode (in red); the second group meant the absolute value of the difference in accuracy between the two types of electrodes is less than 10% (in blue); and the third group meant the accuracy of dry electrode was at least 10% higher than that of the wet electrode (in green). Figure 8b,d shows the distribution of the number of subjects in each group. Most of the subjects had better performance with the wet electrode (FBCCA: 45%, FBTRCA: 50%) and a similar performance with different electrodes (FBCCA: 52%, FBTRCA: 48%). Only a few subjects had better performance with the dry electrodes (FBCCA: 3%, FBTRCA: 2%).

### 3.5. Electrode Impedance and Classification Accuracy

**Figure 9:**
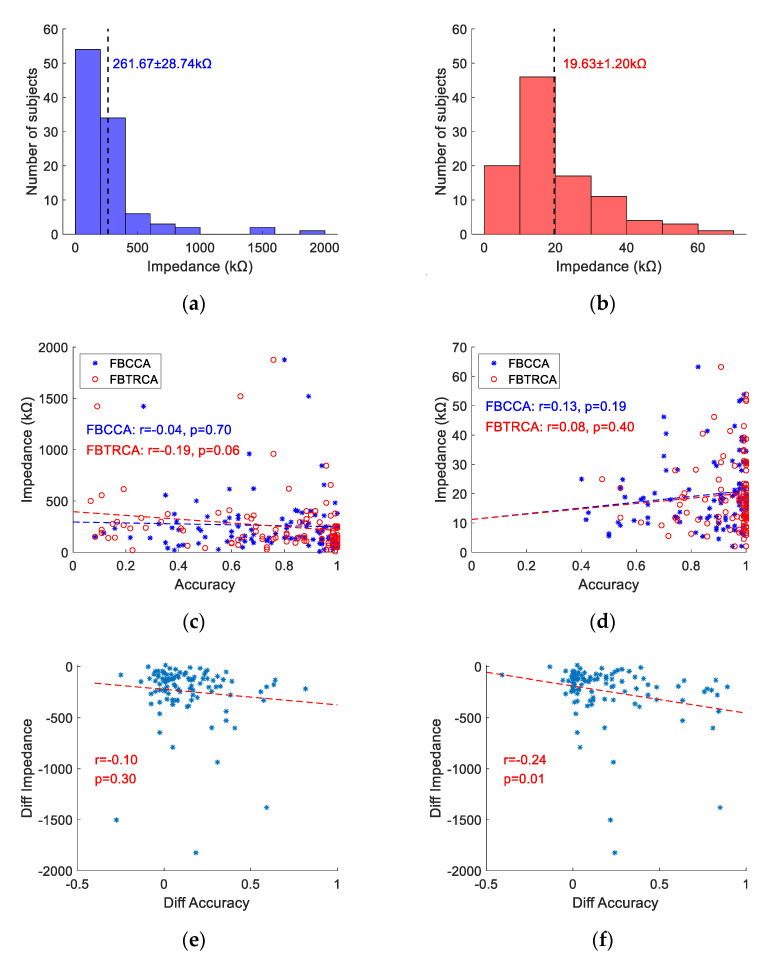
Impedance distribution of (**a**) dry and (**b**) wet electrodes across all subjects. Correlation analysis of the classification accuracy and impedance for (**c**) dry and (**d**) wet electrodes, as well as the accuracy difference and impedance difference between wet and dry electrodes: (**e**) FBCCA, (**f**) FBTRCA. The dotted lines represent the results of linear regression.

The dry and wet electrodes showed different impedance values due to different electrode materials and contact modes with the scalp. Figure 9a,b shows the impedance distribution of the dry and wet electrodes for all subjects, in which the impedance values were averaged across 10 blocks and 8 channels. For most of the subjects, the impedance of dry electrode was less than 500 kΩ and the impedance of wet electrode was less than 40 kΩ, resulting in an average impedance of 261.67 ± 28.74 kΩ for the dry electrode and 19.63 ± 1.20 kΩ for the wet electrode. As shown in Figure 8c,d, there was no significant correlation between classification accuracy and impedance for each electrode (for dry electrode, FBCCA: r=−0.04, p=0.70, FBTRCA: r=−0.19, p=0.06; for wet electrode, FBCCA: r=0.13, p=0.19, FBTRCA: r=0.08, p=0.40).

In order to reveal whether the lower performance of the dry electrode was caused by the increased impedance, this study calculated the difference of accuracy and impedance between the wet and dry electrodes and performed a correlation analysis. As shown in Figure 9e,f, for the FBCCA method, there is a non-significant negative correlation between the difference in accuracy and impedance (r=−0.10, p=0.30). However, there is a significant negative correlation for the FBTRCA method (r=−0.24, p=0.01). These results indicate that less increase of impedance between the dry and wet electrodes will lead to less performance decrease. In other words, higher performance of the dry electrodes can be achieved with reduced impedance of the electrodes.

### 3.6. Information Transfer across Electrodes

By referring to the cross-device method based on the shared latent domain proposed in [15], this study proposed an electrode-transfer (ET) method, which can avoid the time-consuming system calibration when using a new type of electrodes. The sources of SSVEPs were the same for the wet and dry electrodes. Therefore, the SSVEP signals obtained by one type of electrode (train-electrode) can be transferred to the identification method using another type of electrode (test-electrode). For the test-electrode, a spatial filter was found by a least-square method to minimize the sum of squared error between the projected EEG signal of test-electrode and the projected EEG templates from the train-electrode. There was a high correlation between the signals projected by the train-electrode and test-electrode through their respective spatial filters and this correlation coefficient is denoted as $r_{1}$. In addition, the correlation coefficient between the EEG signal of test-electrode and the standard CCA templates is denoted as $r_{2}$. The two feature values can be synthesized as follows:

(5) $$p=sign(r_{1})\times r_{1}{}^{2}+sign(r_{2})\times r_{2}{}^{2}.$$

The correlation coefficients between test data and each target are calculated, respectively, and the stimulus frequency with the highest correlation coefficient value is selected as the target.

**Figure 10:**
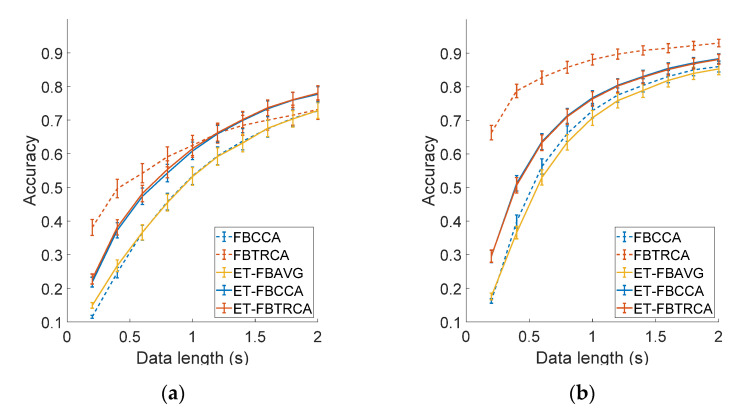
The classification accuracy of the three electrode-transfer (ET) methods and the FBCCA and FBTRCA methods for (**a**) wet-to-dry transfer and (**b**) dry-to-wet transfer. The error bars represent standard errors.

This study adopted a three spatial filtering method to calculate the spatial filter of the train-electrode, including channel averaging (AVG), CCA, and TRCA. These three spatial filters were applied to the ET method and combined with the filter bank analysis to calculate the classification accuracy. The results for the three ET methods (ET-FBAVG, ET-FBCCA, and ET-FBTRCA), FBCCA, and FBTRCA are shown in Figure 10. The optimized parameters of filter banks for the three ET methods were different (wet-to-dry, FBAVG: a=1.5, b=0, N=5, FBCCA: a=2, b=0.25, N=4, FBTRCA: a=1.25, b=0, N=5; dry-to-wet, FBAVG: a=1.25, b=0, N=4, FBCCA: a=1.75, b=0.25, N=4, FBTRCA: a=1.75, b=0.25, N=4). The efficacy of cross-electrode transfer was different for wet-to-dry and dry-to-wet transfers. For wet-to-dry transfer, both ET-FBCCA and ET-FBTRCA were better than FBCCA, while ET-FBAVG and FBCCA were comparable. Moreover, both ET-FBCCA and ET-FBTRCA were even better than FBTRCA when the data length was longer than 1 s (e.g., ET-FBCCA: 0.78 ± 0.02, ET-FBTRCA: 0.78 ± 0.02, FBTRCA: 0.73 ± 0.03 at 2 s). For dry-to-wet transfer, both ET-FBCCA and ET-FBTRCA were better than FBCCA, while ET-FBAVG was slightly worse. However, all ET methods were worse than FBTRCA (e.g., ET-FBCCA: 0.88 ± 0.01, ET-FBTRCA: 0.88 ± 0.01, FBTRCA: 0.93 ± 0.01 at 2 s). These results suggest that information transferred across electrodes can facilitate SSVEP detection, especially when dry electrodes are used to record testing data for BCI applications.

### 3.7. Time-Variant Effects

#### 3.7.1. Questionnaire

All participants filled out the questionnaire on comfort level of the headband, which was divided into four levels (Level 1: comfortable and painless, Level 2: slightly painful, Level 3: painful but acceptable, Level 4: painful and uncomfortable). Higher level indicated more uncomfortable feeling. Table 1 lists the results of the questionnaires. Most participants reported that the wet-electrode headband was comfortable while only a few of them felt comfortable with the dry-electrode headband. Their tolerance time indicates that the subjects’ tolerance to the wet-electrode headband is higher than that of the dry-electrode headband. Some participants also reported “slightly painful” or “painful but acceptable” after wearing the wet-electrode headband for 0.75 and 1 h, respectively. Note that “/” in Level 4 indicates that nobody felt painful and uncomfortable with the wet headband.

Table 2 shows the participants’ preference for the two headbands after the experiment. If only the comfort factor was considered, 83.33% of them preferred the wet-electrode headband, 5.89% preferred the dry-electrode headband, and 10.78% chose either. If the two factors of comfort and convenience (to use conductive paste and wash hair after use) were taken into consideration, the number of participants who preferred the dry-electrode headband increased to 13.72%. However, still 68.63% of the participants preferred the wet-electrode headband. These results indicate that the discomfort of the dry electrode will severely hinder its application in practical BCI systems.

#### 3.7.2. BCI Performance

**Figure 11:**
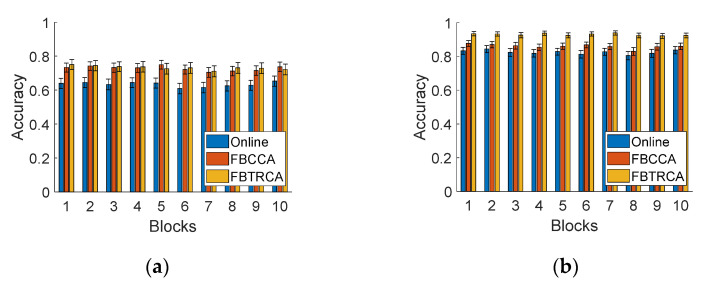

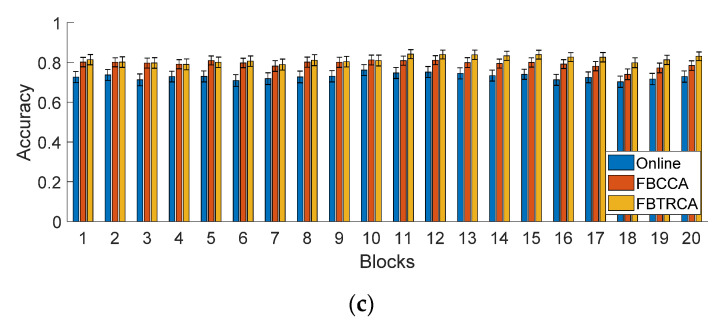
Online and offline (with FBCCA and FBTRCA methods) accuracy with (**a**) the dry-electrode headband, (**b**) the wet-electrode headband, and (**c**) both headbands over all data blocks.

Subjects will feel tired after using the SSVEP-based BCI for a long time, which might affect the performance of BCI. As described in Section 2.5, the participants continued to use the SSVEP-based BCI in a duration of around 2 h with an interval of 15 to 30 min between the tasks with the two headbands. Figure 11 shows the online and offline accuracy of different data blocks over time.

In the experiments, the order of the two headbands was randomized for all subjects. For both of dry and wet electrodes, the accuracy remained stable across the 10 consecutive blocks (Figure 11a,b). Interestingly, even for all 20 blocks, which lasted a total of 1.5 to2 h, there was no clear descending trend of classification accuracy (Figure 11c). These results suggest that a wearable SSVEP-based BCI has stable and reliable performance, which can provide a promising way to implement BCI-based communication and control.

## 4. Conclusions and Discussions

This study provides an open dataset for a wearable SSVEP-based BCI system. Different from other public SSVEP-based BCI datasets, the present dataset used a wearable 8-channel EEG headband to record SSVEPs using an online 12-target BCI paradigm. Importantly, the dataset provided data recorded from wet and dry electrodes from each subject. Besides, the dataset included sufficient data (240 trials for each subject) from a large number of subjects (102 subjects). By analyzing the dataset, we found that the amplitudes and SNRs for wet and dry electrodes were comparable. The classification accuracy reached the highest at 2 s (for FBCCA method, dry: 0.73 ± 0.02, wet: 0.86 ± 0.02; for FBTRCA method, dry: 0.73 ± 0.03, wet: 0.93 ± 0.01), while the *ITR* reached the highest at shorter data length (for FBCCA method, dry: 72.25 ± 5.48 bits/min (1.2 s), wet: 125.6 ± 8.88 bits/min (0.8 s); for FBTRCA method, dry: 201.2 ± 23.87 bits/min (0.2 s), wet: 499.5 ± 27.59 bits/min (0.2 s)). In addition, questionnaires on user comfort were collected from all subjects. Resultingly, 83.33% of the participants felt comfortable with the wet-electrode headband while only 20.59% of them chose the dry-electrode headband. Considering the comfort and convenience factors, 68.63% of them preferred the wet-electrode headband while only 13.72% chose dry-electrode and the other 17.65% thought both were acceptable.

The quality of SSVEP signals was verified by analyzing the temporal waveform, spectral characters and SNR in the dataset. At the same time, the feasibility and practicality of the wearable system were further validated by evaluating the performance of online and offline SSVEP-based BCI. This study also provides a comprehensive comparison of dry electrode and wet electrode in a wearable SSVEP-based BCI system. First, the results of the questionnaires show that although dry electrodes are more convenient to use than wet electrodes, and the more discomfort leads to a lower preference for the dry electrodes. Second, although many subjects can achieve high accuracy with both types of electrodes, the accuracy and *ITR* of dry electrodes are significantly lower than that of wet electrodes. The average SSVEP signals recorded by the two electrodes did not show significant difference among 102 subjects (see Figure 4). The relationship between BCI performance and electrode impedance remains unclear. Third, the detection accuracy can be further improved by optimizing parameters in offline analysis, which suggests that the online algorithms can be further improved in real applications. Fourth, information transfer across different types of electrodes provides a new way to facilitate system calibration. Especially for the dry electrodes, the calibration data from the wet electrodes can lead to higher performance. Overall, dry electrodes need to be further improved to satisfy the requirements of wearable SSVEP-based BCIs for data quality and user experience in practical applications.

In addition to the preliminary analyses provided in this study, the dataset will also play an important and effective role in different aspects for studying SSVEP-based BCIs. First, the data can be easily adopted for developing and evaluating new algorithms for SSVEP detection. Second, efficient noise removal methods and channel selection methods might be helpful for a wearable BCI, especially for the data with the dry electrodes. Third, the dataset can be further used for analyzing BCI demographics due to the large number of subjects.

## Figures and Tables

**Table 1:** Comfort questionnaire results.

	Dry		Wet	
	Proportion (%)	Time (h)	Proportion (%)	Time (h)
Level 1	20.59	0	83.33	0
Level 2	46.08	0.61 ± 0.05	13.73	0.75 ± 0.02
Level 3	13.72	0.76 ± 0.08	2.94	1 ± 0
Level 4	19.61	0.71 ± 0.10	0	/

**Table 2:** Participants’ preference for headbands.

	Comfort (%)	Comfort and Convenience (%)
Dry	5.89	13.72
Wet	83.33	68.63
Either	10.78	17.65

## Data Availability

The data presented in this study are openly available in FigShare at 10.6084/m9.figshare.13560281 or http://bci.med.tsinghua.edu.cn/download.html (accessed on 10 February 2021). We ensure that data shared are in accordance with consent provided by participants on the use of confidential data.
